# Perceptions of a Culturally Tailored Narrative Video for Skin Cancer Prevention Among Spanish-Speaking Hispanic Outdoor Workers: Exploratory Study

**DOI:** 10.2196/98130

**Published:** 2026-08-05

**Authors:** David Perez, Valeria Gómez, Dariana Sedeño-Delgado, Carlos Orellana García, Melida Chacon, Miguel Gonzalez, Chiranjeev Dash, Roxanne Mirabal-Beltran, Alejandra Hurtado-de-Mendoza

**Affiliations:** 1 Department of Oncology, Georgetown University Medical Center, 2115 Wisconsin Avenue, Suite 300, Washington, DC, 20007, United States, 1 2026870100; 2 Epidemiology & Population Science, Baylor College of Medicine, Houston, TX, United States; 3 Catholic Charities, Washington, DC, United States; 4 We Are CASA, Wheaton, MD, United States

**Keywords:** Hispanic outdoor workers, skin cancer prevention, health communication, narrative education, disparities

## Abstract

**Background:**

Outdoor workers receive significantly more UV radiation exposure than indoor workers, increasing their skin cancer risk. Hispanic individuals comprise a large proportion of the outdoor workforce in the United States, but have limited access to culturally tailored prevention resources.

**Objective:**

This exploratory study assessed the perceptions of a culturally tailored narrative video to promote skin cancer prevention among Spanish-speaking Hispanic outdoor workers.

**Methods:**

A qualitative study was conducted using two focus groups with Hispanic outdoor workers. Prior to viewing the video, participants completed surveys assessing sociodemographic factors, sun exposure, and protective behaviors. Focus group discussions explored video comprehension, cultural relevance, emotional engagement, and dissemination strategies. Quantitative data were summarized descriptively, and qualitative data were analyzed using rapid qualitative analysis.

**Results:**

Among 19 participants, the mean age was 46.5 (SD 15.5) years, and 94.7% (18/19) identified as male. While 47.4% (9/19) reported 6‐9 hours of outdoor work per day, more than half (10/19, 52.6%) reported not engaging in sun protection. Participants described the video as culturally resonant, realistic, and engaging. Identification with characters and family-centered themes increased perceived relevance and message credibility. Many participants reported increased awareness of skin cancer risk and greater confidence in adopting sun-protective behaviors. Dissemination recommendations included workplaces, social media, schools, daycares, clinics, and other community settings.

**Conclusions:**

Our video shows promise in improving skin cancer awareness and prevention, reflecting strong acceptability among participants. Future randomized controlled studies are needed to assess its effectiveness in enhancing long-term sun-protective behaviors.

## Introduction

Skin cancer continues to be the leading cancer diagnosis in the United States [1,2]. Basal cell carcinoma (BCC) is the most common form of skin cancer, followed by squamous cell carcinoma (SCC), while invasive melanoma is the deadliest form of skin cancer [3,4]. Each year, there are approximately 3.6 million cases of BCC diagnosed in the United States, and an estimated 1.8 million cases of SCC [4]. Within the past decade, from 2016 to 2026, the incidence of invasive melanoma has increased by 46.6% [2,5,6]. In 2026 alone, it is estimated that 234,680 melanoma cases will be diagnosed in the United States, of which 112,000 will be invasive [2,5]. These projections represent a 10.6% increase in cases and a 1% increase in mortality compared with 2025 [2,5]. UV radiation (UVR) from the sun is widely recognized as the primary risk factor for the development of skin cancer [2,7].

Sunburns, which result from prolonged and intense UVR exposure without sun protection, are reported to be directly associated with an increased risk of both nonmelanoma (BCC and SCC) and melanoma skin cancer [8]. Severe sunburns are among the strongest predictors of melanoma, with evidence showing that even 1 sunburn every 2 years can triple the risk [9,10]. Outdoor workers receive up to 8 times more UVR exposure than indoor workers and have a 60% greater risk of developing skin cancer [11].

Construction and landscaping are among the occupations with the highest proportion of outdoor work time [12]. Despite this, prior interventions targeting these outdoor sectors in the United States remain scarce [13-15].

Interventions for US outdoor workers in the construction and landscape industries are critically needed, especially for Hispanic workers, who represent 35% and 47% of the construction and landscape workforce in the United States, respectively [16]. Although Hispanic individuals have lower melanoma incidence rates than non-Hispanic White individuals, incidence among Hispanic individuals in the United States has increased by 20% in the past 2 decades [17]. Hispanic individuals also experience poorer melanoma outcomes as they are more likely to be diagnosed with thicker melanomas at more advanced stages and have lower melanoma-specific survival rates than non-Hispanic White individuals [17]. These disparities may be particularly relevant among Hispanic outdoor workers due to their increased occupational UVR exposure. Prior research among US Hispanic outdoor laborers (N=175) found that more than half reported experiencing at least 1 sunburn during the summer preceding the study [18]. Hispanic individuals also report low levels of sun-protective behaviors and self-skin examinations [18-20]. Cultural beliefs and social norms play an important role in shaping perceptions of sun exposure risk among Hispanic populations [20,21]. These cultural factors, along with others, such as limited knowledge and awareness, low health literacy and numeracy, limited access to health care, language barriers, and competing priorities, further complicate efforts to promote effective sun protection within this population [20,21].

While recent evidence suggests educational and multicomponent interventions are promising strategies to enhance sun-protective behaviors [14,22], past studies have mostly included workers with fair skin types and were often conducted outside the United States [14,23]. Narrative approaches have emerged as a promising strategy in health communication given their ability to increase engagement and their adaptability across diverse populations [24,25]. Narrative health education materials integrate health information into character-driven stories, unlike traditional didactic materials, which are driven by facts and statistics [26,27]. Narrative communication is hypothesized to influence behavior through multiple psychological processes, including narrative transportation and engagement [28]. Narrative transportation refers to the degree to which individuals are cognitively and emotionally immersed in a story [28]. In addition to decreasing resistance and increasing receptivity to the health message, experiencing “transportation” into the story can support engagement and increase understanding as well as interest in preventive behavior [28,29]. Narrative interventions have also been shown to influence behavioral determinants like self-efficacy by depicting protective behaviors in relatable characters and contexts [30,31]. This may help bridge the gap between knowledge and action by making recommended behaviors clear and feasible [31]. Narratives can also elicit emotional responses that may enhance the impact of the message and motivate behavioral change [32]. Emotion-focused models of health communication suggest that these emotional reactions affect how people pay attention to, understand, and seek information, making it feel more relevant and supporting intentions to change behavior [32].

Evidence indicates that narratives are effective cancer communication approaches among Hispanic individuals [33,34]. Additionally, research has shown that Hispanic outdoor workers prefer videos over printed brochures or fact sheets [35-37]. Most psychoeducation interventions, however, have used a traditional didactic approach, and only a limited number have been developed for Hispanic populations, with even fewer specifically tailored for Hispanic outdoor workers [14,22]. In response to these gaps, we developed a theoretically-informed character-driven video in an effort to increase sun-protective behaviors and promote skin cancer prevention among Hispanic-identifying outdoor workers [38]. To the best of our knowledge, our video is among the first Spanish-language narrative videos tailored for and developed in collaboration with Hispanic outdoor workers living in the United States. This exploratory study aimed to evaluate the perceptions of our culturally tailored narrative education video among Spanish-speaking Hispanic outdoor workers for skin cancer prevention using a qualitative approach.

## Methods

### Overview

To develop the video, we collaborated with a diverse multistakeholder advisory board (MAB) and a community advisory board that included Hispanic outdoor workers, patients, clinicians, and representatives from local and national advocacy organizations. The initial draft of the script was informed by formative focus group findings [20] and developed by the National Conservatory of Dramatic Arts in Washington, DC. Two rounds of script review were conducted, and community advisory board meetings preceded MAB meetings to support Spanish-language discussion and ensure community perspectives informed subsequent MAB deliberations. The finalized script was then translated into Spanish and reviewed by a Spanish-speaking dermatologist on the MAB for production by the National Conservatory of Dramatic Arts. A detailed description of this process is reported elsewhere [38].

Consistent with Kreuter et al [39] framework for culturally tailored health communication, the video included both superficial and deep structure cultural elements. Superficial tailoring was achieved with Hispanic actors, Spanish language dialogue, and culturally familiar elements such as traditional foods and soccer [39]. Deep tailoring targeted cultural values and beliefs identified as salient within Hispanic communities like familismo by depicting family relationships and support as important motivators to engage in sun-protective behaviors and early detection [39,40]. To reflect these tailoring strategies, the video portrays a multigenerational family who owns a landscaping company. The main character, Miguel, is diagnosed with BCC as he is transitioning into taking the lead of his father’s business. The diagnosis propels him and his wife, Sofia, to take an active role in skin cancer prevention at home and in the workplace. Throughout the story, the video provides information about skin cancer, its signs, protective behaviors, and the importance of early detection. The video also models sun-protective behaviors and highlights their significance in all generations. A scene-by-scene description of the video and their associated theoretical constructs are reported elsewhere [38].

To evaluate perceptions of the video, 2 in-person focus groups were conducted between October and November 2025. We recruited participants through trusted community organizations serving Hispanic communities, including Catholic charities and We Are CASA, both of which have established relationships with outdoor workers employed in construction, landscaping, and agricultural occupations. Participants were eligible for this study if they self-identified as Hispanic, were aged ≥18 years, and had current or prior outdoor work experience. Given the focused scope of the study, consisting of focus groups with a targeted sample, data collection was considered complete once data sufficiency was achieved across the focus groups. This was reflected in the convergence of participant responses across sessions with limited emergence of new viewpoints in the second group. Additionally, as this was an exploratory study designed to guide the development and improvement of a future randomized trial, the primary objective was to assess participants’ initial perceptions and potential areas for intervention optimization instead of achieving broad generalizability. Practical factors related to budget deadlines also played a role in the decision to limit the number of focus groups.

### Measures

#### Sociodemographics

Participants were asked to report on their age, gender, race, country of birth, number of years residing in the United States, marital status, educational attainment, health insurance status, health insurance type, annual income, and employment status.

#### Occupational Sun Exposure and Protection Behaviors

Occupational sun exposure and protection behaviors are listed as follows:

1. History of outdoor work: Participants indicated the outdoor occupational sectors they have experience in, including construction, landscaping, agriculture, and/or other.
2. Years of outdoor work experience: Participants recorded their years of outdoor work experience: less than 1 year, 1‐5 years, 6‐10 years, 11‐15 years, 16‐20 years, or more than 20 years.
3. Occupational sun exposure: Participants reported the typical number of hours they work outdoors per day. Responses were categorized as: 1‐3 hours, 3‐6 hours, 6‐9 hours, and 10 or more hours.
4. Sun protection use**:** Participants answered a binary (yes or no) item asking if they engaged in any sun-protective behaviors.
5. Sun protection practices: participants indicated which sun protection strategies they used, if any. Behaviors included wearing sunscreen, a hat, sunglasses, long-sleeved shirts, and long pants.
6. Protection frequency: Participants reported on how often they protect their skin from the sun. Response options included: never, rarely, sometimes, often, or always.
7. Shade time: Participants indicated the amount of time they typically spend in the shade during work. Answer choices included never, rarely, sometimes, often, or always.

#### Focus Group Guide

The focus groups aimed to gather feedback from Hispanic outdoor workers on our culturally tailored video designed to increase skin cancer awareness and prevention strategies. Discussions began with introductions and ice-breakers to create a comfortable environment and establish focus group norms, emphasizing confidentiality and respectful dialogue.

Guided by the Learner Verification and Revision Framework [41], participants were then asked about their perceptions on comprehension, self-efficacy, attraction, and cultural-linguistic acceptability. To further capture narrative-specific mechanisms of engagement, per narrative transportation frameworks [42], participants also reported on emotional engagement, character identification, and transportation. The final section of the guide focused on feasible ways to disseminate the video within their community, including preferred platforms and settings.

#### Quantitative Data Analysis

Descriptive analysis was conducted to summarize sociodemographic and occupational sun exposure and protection behaviors using Statistical Product and Service Solutions (version 31; IBMorp).

#### Qualitative Data Analysis

We analyzed focus group responses using rapid qualitative data analysis, as this method aligns with standard qualitative techniques and is optimized for short, structured studies with defined goals [43]. Using recommended rapid qualitative data analysis practices [43], the analysis was performed in six steps. In step 1, two members of the research team independently analyzed a single focus group transcript and created a neutral domain summary template based on the focus group topics and questions. In step 2, the analysts met to discuss their summary templates and created a consolidated version of the summary template. In step 3, each analyst independently summarized both focus group transcripts using the summary template. In this summarization phase, the analysts developed descriptive summaries consisting of bullet statements organized by domains and identified key quotes, with the goal of providing a concise summary of the data while avoiding interpretation. In step 4, the analysts compared their focus group summaries and refined them through group discussion. In step 5, the analysts compiled the focus group summaries into a matrix organized by domains. In step 6, the team used the matrix to discuss patterns, connect domains, and develop themes.

### Ethical Considerations

This study was reviewed by the Georgetown University Institutional Review Board and granted an exemption determination (IRB #STUDY00007990; exempt under category (2) (ii): tests, surveys, interviews, or observation, low risk). Trained research staff reviewed the informed consent form and study details with participants prior to each focus group, and verbal consent was documented by the study team before any data collection began. Participation was voluntary, and participants were free to decline or withdraw at any time. Data are coded rather than fully deidentified. Focus group audio recordings were transcribed and deidentified for analysis, while any files containing identifying information are stored separately in Georgetown University's secure, password-protected box repository, accessible only to authorized study team members. All participants received a US $50 gift card as compensation for completing the focus group.

## Results

### Sociodemographics

Of the 20 participants recruited, 19 met our inclusion criteria and were included in the final sample. The mean age was 46.5 (SD 15.5) years. Most (18/19, 94.7%) participants were male, and almost half (9/19, 47.7%) identified as “Other” for race. More than half (10/19, 52.6%) reported an education of middle school or less, 57.9% (11/19) indicated an annual income of US $10,000-$39,999, and 63.2% (12/19) did not have health insurance (Table 1).

**Table 1.** Sociodemographics (n=19).

Variable	Values
Age (years), mean (SD)	46.5 (15.5)
Gender, n (%)	
Men	18 (94.7)
Women	1 (5.3)
Race, n (%)	
White	2 (10.5)
Multiracial	1 (5.3)
Other	9 (47.4)
Prefer not to respond	4 (21.1)
Unknown	3 (15.8)
Country of birth, n (%)	
El Salvador	6 (31.6)
Peru	5 (26.3)
Colombia	1 (5.3)
Guatemala	1 (5.3)
Honduras	1 (5.3)
Costa Rica	1 (5.3)
Nicaragua	1 (5.3)
Venezuela	1 (5.3)
Other	1 (5.3)
Unknown	1 (5.3)
Number of years residing in the United States, mean (SD)	10.8 (8.4)
Marital status, n (%)	
Married/living with domestic partner	6 (31.6)
Never married	5 (26.3)
Divorced	1 (5.3)
Other	5 (2.3)
Prefer not to respond	2 (10.5)
Educational attainment	
Elementary school (through 5th grade)	5 (26.3)
Middle school (6th-8th grade)	5 (26.3)
High school diploma/GED^a^	4 (21.1)
Some college	3 (15.8)
Bachelor’s degree	1 (5.3)
Postgraduate degree	1 (5.3)
Annual income, n (%)	
Less than US $10,000	3 (15.8)
US $10,000-$39,999	11 (57.9)
US $40,000-$79,999	2 (10.5)
Prefer not to respond	2 (10.5)
Unknown	1 (5.3)
Health insurance, n (%)	
Yes	7 (36.8)
No	12 (63.2)
Health insurance type, n (%)	
Private (individual)	3 (15.8)
Medicare	2 (10.5)
Medicaid	1 (5.3)
Employer	1 (5.3)
Unknown	12 (63.2)
Employment status, n (%)	
Full-time	6 (31.6)
Part-time	8 (42.1)
Unemployed	4 (21.1)
Student	1 (5.3)

a GED: General Educational Development.

### Occupational Sun Exposure and Protection Behaviors

The majority (8/19, 42.1%) of participants reported 6‐10 years of outdoor work experience. Nearly half (9/19, 47.4%) work 6‐9 hours per day in the sun, and 52.6% (10/19) do not engage in sun-protective behaviors (Table 2). Wearing long-sleeved shirts (13/19, 76.5%) and long pants (12/19, 70.6%) were the most reported sun-protective behaviors, while sunscreen use (4/19, 23.5%) was the least reported (Table 2).

**Table 2.** Occupational sun exposure and protection behaviors.

Variable	Participants, n (%)
History of outdoor work^a^	
Construction	16 (84.2)
Landscaping	9 (47.4)
Agriculture	2 (10.5)
Other	3 (15.8)
Years of outdoor work experience	
Less than 1 year	2 (10.5)
1‐5 years	2 (10.5)
6‐10 years	8 (42.1)
11‐15 years	6 (31.6)
16‐20 years	0 (0.0)
Over 20 years	1 (5.3)
Daily hours working under the sun	
1‐3 hours	3 (15.8)
3‐6 hours	4 (21.1)
6‐9 hours	9 (47.4)
10 hours or more	2 (10.5)
Unknown	1 (5.3)
Use of sun protection	
No	10 (52.6)
Yes	9 (47.4)
Frequency of sun protection	
Never	3 (15.8)
Rarely	2 (10.5)
Sometimes	7 (36.8)
Often	1 (5.3)
Always	6 (31.6)
Amount of time spent in shade during work	
Never	2 (10.5)
Rarely	5 (26.3)
Sometimes	11 (57.9)
Often	1 (5.3)
Always	0 (0.0)
Types of sun protection used^a^	
Long-sleeved shirt	13 (76.5)
Pants	12 (70.6)
Hat	10 (58.8)
Sunglasses	6 (35.3)
Sunscreen	4 (23.5)

a Could select more than one response.

### Focus Group Findings

#### Satisfaction

Participants appreciated the video’s focus on skin cancer, a topic they knew little of and were rarely taught about at work, despite their increased UV exposure:

*I really like it because I was in construction for more than 20 years, and I don’t remember–never–that anyone gave me a talk about the damage that can come from so much sun exposure…and very little is said about skin cancer.*

[Focus group (FG) 1]

Others noted that it completely altered their viewpoint:

It changed mine 100%*.*

[FG 1]

Several described it as credible and trustworthy, stating that the information appeared up to date and was professionally presented.

#### Comprehension and Increased Knowledge

After watching the video, participants reported feeling significantly more knowledgeable regarding skin cancer. Although the video presented substantial information, many participants described it as well-organized and easy to understand:

*I feel that, for me, the message is quite clear. Very clear. Why? Because it talks about what skin cancer is, what the causes are, and what possible actions need to be taken to prevent it*

[FG 1]

Participants were particularly surprised that skin cancer could develop decades after unprotected sun exposure, believing that it happened as late as 1 or 2 years of being under the sun. Participants were also shocked to learn that skin cancer could develop in young and middle-aged adults as a result of early-life UV exposure. As one participant stated:

*To me it seemed very important since I thought that I did not have the capacity to suffer from cancer at an early age. I used to say that only happened to old people, but the information you are giving us seemed very interesting to me, as it explains to us that we are exposed to radiation like ultraviolet rays at all times and we need to protect ourselves.*

[FG 1]

#### Self-Efficacy

Participants especially valued that the content was actionable. When asked how confident they felt in implementing the strategies presented after watching the video, participants expressed strong confidence:

*Compared to before, I’m very confident now.*

[FG 1]

In addition to increasing their confidence and motivation to practice sun-protective behaviors, including using sunscreen, seeking shade, and wearing protective clothing, participants also expressed a strong desire to share this knowledge and empower their loved ones:

*The most important thing is that, after watching this video, I now have more information for myself to take better care of myself. Even though this isn’t currently mandatory at my company, I know I will do it. I will try to take better care of myself, and of course, care for those closest to me as well. I am very grateful for this.*

[FG 1]

#### Attraction

Most participants expressed a clear preference for the narrative story format over didactic explanations delivered by a physician. They reported that the video was engaging and entertaining, capturing their attention without feeling “boring” or overly formal. Many noted that the pacing and length were appropriate, covering all necessary topics without feeling rushed or incomplete:

*I found the video very informative, and I would neither remove nor add anything*

[FG 2]

#### Cultural Relevance and Identification With Characters

Participants also expressed strong identification with the characters. Many commended the video’s portrayal of the close-knit family dynamic representative of Hispanic culture:

*The family support, being with family, home, and all of that is very much directed toward our community.*

[FG 1]

Several particularly valued the story’s themes of friendship, trust, and support for one another, highlighting the trust between Miguel and Sofia as partners and the supportive relationship between Miguel and his father. Participants additionally appreciated the video’s attention to how crucial it is for coworkers to share information in order to protect one another:

*I really liked the support as well, that among the workers there is support, and the knowledge is shared with others so they don’t fall into the same mistake as someone else*.

[FG 2]

The video was described as very relevant to their daily work and life experiences. Many emphasized how realistic and representative the characters’ work experiences felt compared to their own lives. In addition to capturing their daily realities, several expressed that the video effectively clarified widespread myths among Hispanic individuals regarding sun protection and the risk of skin cancer. Participants specifically noted that it competently addressed machismo*,* a key cultural barrier to sun-protective behaviors among Hispanic men characterized by traditional masculinity, by alluding to the cultural influence of soccer players in practicing sun-protective behaviors (eg, sunscreen use). Many also appreciated that the video was produced in Spanish as it not only felt like it spoke to them directly but also addressed another key barrier for those with low literacy. According to one participant:

*I think it is very focused on the position of the Hispanic worker. Obviously the language, the physical appearance, and all those qualities that represent us are very evident.*

[FG 2]

#### Emotions

Participants further described feeling engaged and immersed in the storyline from start to finish. Several mentioned that the video evoked strong emotions like sadness and concern, particularly when thinking about how a cancer diagnosis could affect a person’s family. One participant shared the emotional impact:

*I was about to cry. [Laughs] I looked down twice so they [other focus group participants] wouldn’t see me.*

[FG 1]

At the same time, participants liked that the video ended on a hopeful note, focusing on self-agency and prevention:

*I’m thankful, as she [other participant] said, to let family know that there is a way to treat cancer. There are those of us who say, ‘We go through it, and at that point, I’m going to die, I’m going to die.’ But always, at the end of the road, there is always a way out. Thank you.*

[FG 1]

This transition allowed participants to acknowledge the risks of sun exposure while feeling encouraged to take actionable steps.

#### Suggestions for Improvement

To enhance the effectiveness of the video, participants suggested having the video presented by a professional available to answer questions and pairing it with a pamphlet to include (1) listing providers they could contact and locations where they could seek treatment if needed, (2) additional information on sunscreen types and application, and (3) providing illustrations of how skin cancer can appear across different skin tones and ages. As one participant expressed:

*The information I got from watching the whole video about the doctor—that he [Miguel] has been well cared for—was before things progressed further. But I think that, like all of us Latinos [used interchangeably with the term “Hispanic” at a similar frequency], there’s something here in this country that we fear even more than cancer itself. I mean, going to a doctor. We think, if we go to a doctor, how much is it going to cost me? What will the bill be? And because of that fear, we don’t get treated; we just let it go. So, I think that including a reference, that is, a phone number where you can contact this doctor, or this person, someone who can see you, or something along those lines would be helpful.*

[FG 2]

### Implementation and Dissemination Strategies

#### Barriers to Implementation

Participants identified several barriers related to implementing sun-protective behaviors and sharing the video. Personal factors, like forgetting sunscreen, past allergic reactions, and occasional inconvenience, were mentioned as potentially hindering protective behaviors. Occupational barriers included lack of company support for sun protection, absence of formal orientation or training, and work uniforms that do not provide enough coverage. As expressed by one of the participants:

*There’s definitely a lack of education in this area, right? A huge lack of education when it comes to sun protection, and clearly companies don’t care because they’re focused on production. I mean, they need production, so it’s not going to matter to them. Like, ‘look, let’s stop the equipment—right now the sun is so hot, we’re going to shut it down for half an hour or an hour so the workers can rest and hydrate a bit.’ That generally doesn’t happen, and it doesn’t happen either because the necessary education isn’t there, or because they really don’t care about anything beyond production.*

[FG 1]

#### Facilitators to Implementation

To address these barriers, participants suggested strategies to enhance awareness. One participant explained:

*I think the first step is to give informative talks about this situation and make them [outdoor companies] aware of the purpose, the importance of what we’re discussing, and how to protect the worker in their own defense.*

[FG 2]

Participants also recognized the importance of self-agency in sun protection, emphasizing that individuals cannot rely entirely on employers to provide guidance or resources. Cost was generally not viewed as an obstacle when sunscreen was seen as helpful and effective. Participants mentioned that clear and culturally relevant messages, identification with characters, understanding of risks, and support from family were significant factors that encouraged behavior change. They also noted that the confidence gained from watching the video would motivate them to use protective strategies.

#### Suggestions for Dissemination

To increase the video’s impact and reach, participants offered several suggestions. They advised disseminating it via social media sites like YouTube (Google LLC) and Facebook (Meta Platforms, Inc), as these platforms are accessed by individuals of all ages, including children:

*Now I think there is a lot of technology, and I believe that is what we are most focused on—social media. Often, I think we can use it as a way to inform people, because nowadays even the youngest children have Facebook, Instagram, and other platforms. So perhaps using social media could be an effective way to raise awareness*

[FG 1]

Participants also suggested sharing it within workplaces, especially during orientations or preshift meetings (eg, toolbox talks). Use of QR codes and links sent by companies, along with follow-up question-and-answer sessions, were also encouraged to not only ensure engagement, but also comprehension.

Broader dissemination strategies included incorporating the video into community settings. Participants particularly emphasized the importance of introducing the video early, such as in daycare and schools, so that awareness of skin cancer risk and sun-protective behaviors can be established from a young age. Other sites such as clinic waiting rooms were also encouraged so viewers could further discuss any thoughts or questions with their clinicians.

Many participants also expressed strong interest in having resources like these acknowledged and supported by occupational safety campaigns as well as organizations such as the Occupational Safety and Health Administration. According to one participant:

*I believe that, for there to be education about skin cancer, the first focal point should be OSHA because it is the largest department here in the United States, and many Latin American countries, as well as other parts of the world, also follow its guidance.*

[FG 1]

It was additionally suggested that the video be shared by, or shown in the presence of, a skin cancer survivor, as this could help viewers relate the narrative to real-life experiences and increase its impact. Participants also emphasized the importance of timing, recommending that sharing the video during the summer months, when sun exposure is highest, would be particularly important. See Tables 3 and 4 for additional quotes.

**Table 3.** Video reactions.

Category	Summary	Quotes
Satisfaction	Appreciated that the video discusses a topic that isn’t taught about despite their increased risk as outdoor workers Highly satisfied with the video’s explanation of skin cancer and the steps they can take to address it Liked that the information is up to date	“I think that, as [name] says, the video is very good, so that we can learn what skin cancer causes, its consequences, and how to correct it.” (FG^a^ 1) “I think the video is good; it is clear with the information it has, and it is up to date with the current times.” (FG 2)
Comprehension and increased knowledge	Found the video very easy to understand Clear message and sequence of events Increased understanding of long-term risks of UV exposure Didn’t know skin cancer could happen up to 20 years after UV exposure Weren’t aware of the UV index Misconceptions about UV susceptibility in the Hispanic community	“It is very interesting how the video presented the topic, and with very, very clear language, which I think we all should know, and we should also share it with our family, and later with our work groups.” (FG 1) “I was surprised by something they mentioned there that I really had no idea about, that this cancer could appear 5, 10, even up to 20 years after having been exposed to the sun. I thought it was something earlier, right? I thought it could be something like 1 year, 2 years, or probably 3.” (FG 1) “I thought it was very important because I thought that because I am young, I didn’t have the chance of getting cancer at an early age. I used to say that that only happened to old people, but I found the information they are giving us very interesting, because it makes us aware that we are exposed to radiation like ultraviolet rays at all times, and we need to protect ourselves against it.” (FG 1) “I think that what catches my attention is that people think that because of their color they are not exposed to harmful damage, and that is something that gets instilled in you from when you are little.” (FG 2)
Self-efficacy	Felt more confident applying sun-protection strategies learned from the video Greater motivation to implement behaviors and share protection strategies with family members	“After watching the video, how confident are you that you can follow its recommendations?” “100 %.” (FG 2) “I’m going to take it very much into account and share it with my family and all that. I didn’t know about this.” (FG 2)
Attraction	Story format preferred over didactic explanations by a physician Length and pacing were appropriate as the video covered all necessary topics without feeling rushed Wouldn’t add or take away anything	“It’s not long and it’s complete. It gives the information—the process of cancer, how to treat it, and how to prevent it, which is what’s important.” (FG 2) “The story resonated with me more because the doctor explains things in a way that works well.” (FG 2) “I think the video is well structured and has a sequence.” (FG 1)
Cultural relevance and identification with characters	Reflects real workplace dynamics and experiences Appreciated that the video is in Spanish Family support portrayed is heavily representative of Hispanic culture Effectively explores cultural gender norms (eg, machismo) and offers an innovative approach to their associated barriers Many participants expressed a strong desire to share the video with coworkers and family Characters look and speak like them Liked the trust shown between Miguel and Sofia as partners Identified with Miguel and his father due to their strong family dynamic Enjoyed the theme of companionship and information-sharing within the company	“I really identify a lot with the video because in my particular case, I am someone who works almost always exposed to the sun, and I think it has been like that for a long time. I’ve had jobs where I spend a lot of time outside.” (FG 1) “I thought the video was very good. Especially directed toward the Hispanic community.” (FG 2) “How much do you think the video reflects the Hispanic community? Or how much do you think it can resonate with the Hispanic community? I think 100 %. Yes, 100 %.” (FG 1) “They were very well acted; they seemed like professionals.” (FG 2) “I really liked the trust between the couple because it says that between partners it is mutual to help each other.” (FG 2) “We Hispanics are like them—when someone says, ‘Hey, put on sunscreen,’ the response is often, ‘Man, no, that’s not for us.’ As we say, ‘hágale huevo,’ meaning we just push through and act tough. The video portrayed this well, showing how people typically resist, saying, ‘No, we don’t use that, just do it this way,’ which makes it relatable and understandable.” (FG 1)
Emotions	Evoked sadness about potential consequences of skin cancer Ended on a positive note, leaving participants with a sense of empowerment Emotional scenes with family were impactful and culturally resonant	“Sometimes we don’t take care of ourselves, or we don’t take it seriously, and in the end, it ends up harming us.” (FG 2) “I felt good because everything ended positively.” (FG 1)
Suggestions for improvement	Include resources in a pamphlet about where they can seek treatment if needed Have a professional available to answer questions that may arise during the video Testimony from a real individual with melanoma could enhance motivation Emphasize legal advocacy and integration (eg, OSHA^b^ regulations) More information on sunscreen application and type Include images of skin cancer across a range of skin colors and ages	“The final point, where I think it is important to reach, is that besides having awareness about skin cancer, it is also about integrating it into OSHA’s safety system for work. Well, it has always been talked about only using helmets, glasses, gloves, and it would also be very good to adopt the issue of skin protection, which is never talked about.” (FG 1)

a FG: focus group.

b OSHA: Occupational Safety and Health Administration.

**Table 4.** Implementation and dissemination strategies.

Category	Summary
Barriers to implementation	Hispanic individuals are not accustomed to using sunscreen compared to non-Hispanic individuals Most companies do not teach about sun protection or provide resources for it Easy to forget sunscreen Hispanic men often neglect self-care Past allergic reactions to sunscreen Can be inconvenient during hot days
Facilitators to implementation	Recognize the importance of self-agency and not relying entirely on employers Cost of sunscreen is not an issue if it is useful and effective Clear and culturally relevant messaging Knowledge of risks and long-term consequences Confidence gained after watching the video Mobile-friendly
Suggestions for dissemination	Worksite orientations and training QR codes Social media (eg, YouTube, Facebook, and Instagram) On-site informational community sessions Schools and daycares Clinics, contractors, and public organizations focused on occupational safety (eg, OSHA^a^) Involve skin cancer survivors Increased promotion during the summer months

a OSHA: Occupational Safety and Health Administration.

## Discussion

### Principal Findings

This study strongly supports the potential of a culturally tailored narrative video for skin cancer prevention in engaging Hispanic outdoor workers, supporting comprehension, and increasing confidence in adopting preventive behaviors. Building on prior work on narrative interventions in cancer education [44-47], this study addresses a critical gap in skin cancer prevention and represents one of the first reported narrative videos tailored for and developed with Hispanic outdoor workers, providing early insights into its perceived potential.

Consistent with prior research, our findings emphasize the importance of interventions that reflect the cultural values and lived experiences of the intended audience [48,49]. Participants repeatedly noted that they appreciated the video being presented in Spanish as it not only strengthened its cultural connection but also addressed a systemic gap. In addition to highlighting the portrayal of familism as central in character relatability, participants appreciated how the video addressed specific constructs that could hinder sun protective practices in and out of work. Our findings support the notion that narratives can create opportunities for participants to not only see themselves in the story but also envision themselves adopting recommended behaviors [50]. In our study, participants’ connection with Miguel, his father, and other culturally resonant role models, such as soccer players, demonstrates the potency of narratives in addressing culturally specific barriers, including gender norms like machismo, while simultaneously promoting self-efficacy.

Additionally, by causing feelings of concern and introspection, the video appears to have not only enhanced message processing, but also reinforced personal relevance, especially in relation to family members. Although the video showed the potential consequences of skin cancer, participants particularly appreciated that it ended on a positive note. Beyond keeping participants engaged with the narrative, this balance of emotional intensity likely increased their immersion and subsequent self-efficacy, consistent with narrative transportation frameworks [51]. This was reflected in their responses, as many stated that the video had completely changed their perspective and motivated them to start practicing sun-protective behaviors in their everyday lives.

Participants’ recognition of self-agency in sun protection despite limited employer support also highlights the importance of interventions that empower workers to take preventive action. Many participants described situations in which workplace infrastructure and/or lack of formal training made reliance on employers alone insufficient for maintaining sun safety. This focus on self-agency reflects health behavior theories that view individuals as active participants in shaping their health outcomes [52,53]. For example, Social Cognitive Theory emphasizes human agency and self-efficacy as important factors in behavior and highlights that individuals are more likely to engage in protective actions when they perceive they can successfully perform those behaviors even in constrained environments [30,31,52]. Likewise, the Theory of Planned Behavior states that perceived control over behavior is a key predictor of intentions and actions related to health, especially when structural barriers exist [53,54]. Empowerment-oriented approaches add that improving individuals’ perceived control over health decisions is a key mechanism for improving outcomes, particularly in resource-limited settings [55]. Together, these theories highlight the need for interventions that build individual capacity by increasing knowledge and confidence in practicing protective behaviors.

At the same time, our findings also stress the importance of supportive workplace policies that make these behaviors practical and sustainable. Encouraging self-efficacy with structural supports aligns with health behavior theories emphasizing that personal agency is most effective when reinforced by enabling environments [56]. This moreover supports the need for multilevel interventions for employees and employers that combine individual-level strategies with organizational policies and environmental supports to maximize adherence and long-term health outcomes. Participants identified multiple ways in which resources like these could be incorporated into their workday, particularly during orientations or preshift meetings, demonstrating both the feasibility of integrating preventive strategies into routine workflows and participants’ interest in such resources. Importantly, in contexts where employer-provided resources may be limited, participants emphasized that knowledge about the importance of sun protection could best support such practices, as the cost of items like sunscreen would not be a barrier if perceived as beneficial for their health. To further address the individual-level implementation barriers identified (forgetting sunscreen, allergic reactions to sunscreen, etc), increased complementary education regarding electronic reminders and/or alternative sunscreen formulations (eg, hypoallergenic, mineral-based) for sensitive skin could be helpful.

Participants also recommended sharing the video beyond worksites such as schools, daycares, clinics, and across social media platforms. These suggestions reflect not only their positive reception of the video, but also their perception of its broader importance and relevance. For many Hispanic outdoor workers, employment is closely tied to their role as providers. Protecting their skin is not viewed as an individualistic health behavior, but as a means of safeguarding their ability to remain present and provide for their loved ones [21]. Several participants expressed that the video directly speaks to this sense of responsibility. Additionally, after learning that skin cancer most commonly develops over many years, several expressed a desire to ensure that their families and friends across all generations grow up with more accurate knowledge and preventive habits. Overall, the potential of our video is highlighted by the interest and motivation of participants in implementing and sharing the knowledge obtained from the video, particularly in a cohort in which more than half did not engage in sun-protective behaviors.

Beyond these promising findings, this study has several key strengths. First, its qualitative design enabled a thorough exploration of participants’ viewpoints and provided detailed information on experiential aspects that quantitative methods would be unlikely to capture, such as cultural relevance, emotional engagement, and distribution strategies. Second, the study targeted a high-risk group with limited access to culturally-relevant skin cancer prevention resources, not only addressing a significant research gap, but also potentially improving the real-world applicability and impact of the video. Third, it produced practical recommendations to enhance the video’s effectiveness and guide distribution strategies, including perceived facilitators and barriers to implementation. Fourth, the study provides insights that can guide future research, including randomized control trials and multilevel interventions with employers. Despite these strengths, several limitations should also be noted. First, this was an exploratory study with a small sample size (N=19) and a male-dominated sample (18/19, 94.7%), limiting the transferability of the study to other Hispanic outdoor workers. The small sample size also limits the range of perspectives captured and the degree to which subgroup differences are reflected in the findings. Female workers were especially underrepresented, and their views on sun protection knowledge and behaviors, as well as perceived barriers and facilitators, may differ in important ways that were not fully captured, similar to other subgroups. Second, data were self-reported, potentially introducing social desirability bias as well as recall bias. Third, with a focus group format, participants’ answers may have been influenced by other participants.

Future research should more rigorously evaluate the acceptability of the video, including its impact on sustained sun-protective behaviors over time. Randomized controlled trials comparing this narrative approach to standard educational materials, including a larger sample, are needed to determine its effectiveness. Studies involving employer and occupational health engagement could also assess strategies for integrating sun protection into workplace policies. Future studies could further explore whether the impact of the video differs across outdoor work sectors (construction vs landscaping, agriculture vs construction, etc).

### Conclusion

This exploratory study provides early evidence supporting the acceptability and promise of our culturally tailored narrative video intervention to promote skin cancer prevention among Spanish-speaking Hispanic outdoor workers. Feedback regarding its cultural and occupational relevance was strongly positive, with self-reported improvements in knowledge and self-efficacy. Additional research is needed to determine if this approach can meaningfully enhance sun protective behaviors to reduce skin cancer risk among Hispanic outdoor workers at scale.

## Abbreviations

BCC: basal cell carcinoma
MAB: multistakeholder advisory board
SCC: squamous cell carcinoma
UVR: UV radiation
